# Diamond detector in absorbed dose measurements in high‐energy linear accelerator photon and electron beams

**DOI:** 10.1120/jacmp.v17i2.5690

**Published:** 2016-03-08

**Authors:** Ramamoorthy Ravichandran, John Pichy Binukumar, Iqbal Al Amri, Cheriyathmanjiyil Antony Davis

**Affiliations:** ^1^ Medical Physics Unit Department of Radiation Oncology National Oncology Center Royal Hospital Muscat Oman

**Keywords:** diamond detectors, small field RT, absorbed doses, stereotactic radiotherapy

## Abstract

Diamond detectors (DD) are preferred in small field dosimetry of radiation beams because of small dose profile penumbras, better spatial resolution, and tissue‐equivalent properties. We investigated a commercially available ‘microdiamond’ detector in realizing absorbed dose from first principles. A microdiamond detector, type TM 60019 with tandem electrometer is used to measure absorbed doses in water, nylon, and PMMA phantoms. With sensitive volume 0.004 mm^3^, radius 1.1 mm, thickness $1\times 10^{-3}\text{mm}$, the nominal response is 1 nC/Gy. It is assumed that the diamond detector could collect total electric charge (nC) developed during irradiation at 0 V bias. We found that dose rate effect is less than 0.7% for changing dose rate by 500 MU/min. The reproducibility in obtaining readings with diamond detector is found to be ±0.17% (1 SD) (n=11). The measured absorbed doses for 6 MV and 15 MV photons arrived at using mass energy absorption coefficients and stopping power ratios compared well with $N_{d}$, water calibrated ion chamber measured absorbed doses within 3% in water, PMMA, and nylon media. The calibration factor obtained for diamond detector confirmed response variation is due to sensitivity due to difference in manufacturing process. For electron beams, we had to apply ratio of electron densities of water to carbon. Our results qualify diamond dosimeter as a transfer standard, based on long‐term stability and reproducibility. Based on micro‐dimensions, we recommend these detectors for pretreatment dose verifications in small field irradiations like stereotactic treatments with image guidance.

PACS number(s): 87.56.Da

## I. INTRODUCTION

Dosimetry of today relies mainly on ion chambers (IC), silicon diodes, film, and thermoluminescent dosimetry (TLD). All have their own characteristics, advantages, disadvantages, and applications. The dose profile penumbras of the diamond detector are smaller than the ionization chambers, indicating a better spatial resolution. The ‘diamond’ is almost tissue‐equivalent, as ‘diamond’ is purest form of natural carbon, and with atomic number Z=6 which is close to that of tissue (Z=7.4). The ‘mass collision stopping power ratio’ and ‘mass energy absorption coefficient ratio’ of tissue to diamond is almost constant for energies used in clinical applications. Other solid‐state detectors, like silicon diodes, have higher atomic number (Z=14), exhibit more energy dependence, and therefore overestimate the dose at lower energies. The relatively large band gap of 5.6 eV for carbon (diamond) prevents leakage currents in the lattice.

The diamond has high radiation sensitivity and therefore very small detectors are manufactured while keeping good signal statistics. The small size results in good spatial resolution and makes the diamond detector suitable for applications with large dose gradients and small fields, where ion chambers do not have application.

Diamond detector's efficacy has been investigated in linear accelerator 6 MV photon beam^(1)^ with a radiation field analyzer and also its performance was compared with an ion chamber. Dose linearity, dose rate dependence, depth‐dose distribution, as well as dose profiles, demonstrated better dosimetric characteristics for diamond detector. Another study^(2)^ demonstrated that there is insignificant directional dependence in DD (up to±15°the deviation is<1%). The dose profiles of the IC and DD follow each other to a satisfactory extent and the penumbras of the diamond detector are narrower than the ion chamber. This is shown to be due to better spatial resolution of DD due to its smaller active volume.

During demonstration of volume effect of detectors in one study,^(3)^ local discrepancies of more than 10% are found between calculated cross profiles of intensity‐modulated beams and intensity‐modulated profiles measured with film. Absolute dose measurements of intensity‐modulated fields with a 0.6 cm^3^ Farmer chamber showed significant differences of more than 6% between calculated and measured dose values at the isocenter of an IMRT treatment plan. Differences of not more than 2% are found for dose values measured with a 0.015 cm^3^ pinpoint ion chamber. A method to correct for the spatial response of finite‐sized detectors and to obtain the “real” penumbra width of cross profiles from measurements was introduced. Output factor measurements were performed with different detectors and presented as a function of detector size for a $1\times 1\;{\text{cm}}^{2}$ field. Because of its high spatial resolution and water equivalence, a diamond detector was found to be suitable as an alternative to other detectors for small field dosimetry like photographic and photochromic films, TLDs, or water‐equivalent scintillation detectors.^(3)^

In another work,^(4)^ the possibility to use a commercial chemical vapourization‐type diamond detector (CVD detector) for proton beam dosimetry was investigated. Thorough investigation of detector‐ response as a function of dose, dose rate and the priming/rise time showed the suitability of CVD diamond for dosimetry of clinical 62 MeV proton beams. With clinical high‐energy proton beams, the ‘diamond’ response versus absorbed dose and dose rate was very good and better stability (in terms of calibration factors over many weeks of use) within low values of reproducibility (under 3%) was demonstrated. In intensity‐modulated prostate radiotherapy treatments, verification dosimetry measurements made with a diamond detector, an Exradin A12 ion chamber, a PinPoint ion chamber and with portal dosimetry EPID. These measurements brought out efficacy of diamond detector in segmental subfields dose measurements.^(5)^ Theoretical characterization of diamond detectors in particle accelerator beams was outlined in another report.^(6)^

Most recently, some authors^(7)^ investigated the utilization of natural diamonds in small field dosimetry, more specifically for the measurement of total scatter factor for the CyberKnife system and for dose measurements of radiation beams in intensity‐modulated radiation therapy (IMRT), respectively. An analysis of total scatter factor obtained using diamond detector was made, along with data from PinPoint ion chambers and semiconductor diodes. It was also outlined that, in depth‐dose measurements, the dose rate dependence was less than 0.2%.

Reports in the literature have mostly explained the ‘diamond detector’ characteristics, more like a solid‐state detector, but there are not many reports indicating absorbed dose calibrations using ‘diamond detector’ in a tissue‐equivalent medium, for absolute dosimetry. Anew design of microdiamond detector, first of its kind, was acquired by our department. We investigated its application to derive absorbed dose at reference point in water, for linear accelerator radiation beams.

## II. MATERIALS AND METHODS

### A. Detector and electrometer

A microdiamond detector, type TM 60019 (PTW, Freiburg, Germany) along with tandem electrometer is used in this study. This has specifications of long‐term stability per year ≤0.5%; ≤0.25% and ≤0.3% for 1 KGy for 18 MV photons and 18 MeV electrons, respectively. With sensitive volume 0.004 mm^3^, radius 1.1 mm, thickness $1\times 10^{-3}\text{mm}$, the nominal response mentioned in technical manual is 1 nC/Gy. The recommended operating voltage for this design is V=0 volts. Leakage current ≤±20 fA makes it suitable for measuring very small signals.^(8)^

### B. Measurement of absorbed dose

The nominal sensitivity of the microdiamond crystal detector 1 nC/Gy is used to get dose in Gy to the detector surrounded by a medium. The measured dose is converted into absorbed dose to medium using reported values of ‘true mass energy absorption coefficients’ and ‘stopping power ratios’ of the medium with respect to ‘diamond detector’ (graphite or carbon), for the respective energy spectrum under study. To develop a theory to use this diamond detector for absorbed dose, experiments were carried out in water, nylon, and PMMA (Perspex) phantoms.

### C. Calculation of nominal sensitivity

The following calculations show how the sensitivity of the diamond detector is arrived at: $\text{radius}=1.1\;\text{mm}\;(1.1\times 10^{-3}m)$; $\text{thickness}=1\times 10^{-3}\text{mm}\;(1\times 10^{-6}m)$; ${\text{density}}_{\text{diamond}}\;\rho =3510\;\text{Kg}/m^{3}$.

Therefore, mass of the diamond detector is derived as $13.35\times 10^{-9}\text{Kg}$. 1 Gy absorbed dose corresponds to a radiation energy absorption of 1 J/Kg. 1 Gy produces $13.35\times 10^{-9}$ J of absorbed energy in the mass of diamond detector of $13.35\times 10^{-9}\text{Kg}$, which is equal to 1 J/Kg. $13.35\times 10^{-9}$ J corresponds to $8.333\times 10^{10}\;\text{eV}$ of energy imparted.

For diamond, “the average energy needed to produce an ion pair” 13 eV/ion pair.^(6)^ Therefore, $8.333\times 10^{10}\;\text{eV}$ of energy produces $[8.333\times 10^{10}\text{eV}/(13\text{eV}/\text{ion pair})]=6.41\times 10^{9}$ ion pairs. Assuming there is no recombination and entire charges takes part in photoconduction, the amount of charge corresponds to $6.41\times 10^{9}(\text{electrons})\times 1.602\times 10^{-19}(C/\text{electron})=10.269\times 10^{-10}C=1.0269\;\text{nC}$. Therefore, the sensitivity of the detector is 1.0269 nC/Gy. This value is in agreement with the quoted value of nominal sensitivity of 1 nC/Gy by the manufacturer.

### D. Dose rate effect

The signal of the diamond detector (nC) collected for monitor repetition rates 100 MU/min and 600 MU/min at standard geometry in water phantom. The dose rate dependency of the diamond detector sensitivity is explained by Fowler^(10)^ who introduced an expression for the dose rate dependency of the detector linearity, which was quoted by other researchers.^5^, ^11^ The detector signal (S) versus the dose rate $\left(\overset{ˇ}{D}\right)$ is expressed by the equation

(1) $$i=R.{\left(\overset{ˇ}{D}\right)}^{\Delta }+i_{dark}$$

If $\overset{ˇ}{D}=1$, the relationship is linear; *R* is a detector‐specific constant, and $i_{dark}$ is the signal due to the dark current of the detector. This equation for dose rate dependence is used to find Δ, the sublinear dose rate parameter, at 6 MV and 15 MV photon beams from Clinac 2300 CD (Varian Medical Systems, Palo Alto, CA) linear accelerator. The temperature dependence for diamond detector in an earlier report^(2)^ claimed that within the investigated temperatures 13°, 22.5°, and 37°C, the variation in signal was within 1.8%. The value of “the average energy needed to produce and ion pair” 13 eV/ion pair, obtained from earlier reference^(6)^ is at 300° K (which is around the temperature at which the detector is normally used).

### E. Response to scattered photons

At 10 cm depth in water, the diamond detector and ion chamber response was studied for $10\times 10\;{\text{cm}}^{2}$ and $30\times 30\;{\text{cm}}^{2}$ fields for 6 MV and 15 MV photons. To see the response of the diamond detector for small fields, measurements of diamond detector was compared with CC 01 ionization chamber (air volume 0.01 cc) (M/s IBA, Schwarzenbruck, Germany) in Perspex phantom (Imperial Chemical Industries, London, UK), 30 cm×30 cm×15 cm dimensions, detector at 5 cm depth. Smaller field sizes less than 10×10 cm were obtained with microMLC (mMLC)(Brainlab AG, Feldkirchen, Germany) (3 mm MLC septa width at isocenter), attached to Varian 600 CD linear accelerator. As we obtained scatter comparison in Perspex medium, separately the measured values of CC01 were compared with earlier measured values of ‘in water phantom’.

### F. Mass energy absorption coefficients and stopping power ratios

Table 1 shows the values of $\mu_{\text{en}}/\rho$ ratios of water, Perspex, nylon, and carbon (for diamond detector) with respect to air.^(12)^ The calculated values of $\mu_{\text{en}}/\rho$ ratios obtained for water, Perspex, and nylon with respect to diamond detector (carbon) are also shown in the same table. Table 2 shows the average restricted mass stopping power ratios of different materials with respect to air. In the same table, the calculated ratios with respect to carbon are also shown.

**Table 1:** Listed and calculated ratios of mass energy absorption coefficients

*Photon Energy*	(μen/ρ) *water air*	(μen/ρ) *ppx air*	(μen/ρ) *nylon air*	(μen/ρ) *carbon air*	(μen/ρ) *water carbon*	(μen/ρ) *perspex carbon*	(μen/ρ) *nylon carbon*
6 MV	1.111	1.078	1.090	0.997	1.120	1.087	1.099
15 MV	1.105	1.063	1.075	0.986	1.121	1.078	1.093

**Table 2:** Listed and calculated average restricted mass stopping power ratios

*Photon Energy*	(L/ρ) *water air*	(L/ρ) *ppx air*	(L/ρ) *nylon air*	(L/ρ) *carbon air*	(L/ρ) *water carbon*	(L/ρ) *perspex carbon*	(L/ρ) *nylon carbon*
6 MV	1.127	1.093	1.129	1.002	1.125	1.091	1.127
15 MV	1.106	1.074	1.097	0.982	1.126	1.094	1.117

### G. Absorbed dose measurements

Absorbed‐dose measurements were carried out with a $50\times 50\times 50\;{\text{cm}}^{3}$ dimension water phantom (Blue Phantom, IBA), with radiation beams from Clinac 2300 CD and Clinac 600 CD linacs (M/s Varian Medical Systems). The diamond detector was positioned at 10 cm depth, with field size $10\times 10\;{\text{cm}}^{2}$ at surface of the water. The focus surface distance 100 cm. A priming dose of 500 MU was delivered before obtaining electrometer readings. Absorbed dose was calculated using the parameters used in Eq. (2). At same positions, the compact ion chamber 0.13 cm^3^ volume, (CC 13, IBA) was positioned for measurements of absorbed dose, following TRS‐398 IAEA protocol.^(13)^

A cylindrical isocentric tool Perspex (PMMA) check phantom (Sun Nuclear Corporation, Melbourne), having diameter 10 cm, is used to position diamond detector probe at isocenter to compare with two different ion chambers (FC 65, CC13, both calibrated from IBA) by ‘substitution’ method. The focus‐to‐axis distance (FAD) was kept as 100 cm, with depth of the detector center at 5 cm. For 6 MV and 15 MV beams, the dose to Perspex at the center of the phantom derived for diamond detector, D, from first principles using charge, Q, from relation Eq. (3).

(2) $$\begin{array}{l} D{\left(\text{GY}\right)}_{\text{Water}}=Q{\left(\text{nc}\right)}_{\text{water}}\times [1/\text{Sensitivity}\;\text{nC}/\text{Gy}]\times [S_{\text{water}}/S_{\text{carbon}}]\times \\ \;[{\left(\mu_{\text{en}}/\rho \right)}^{\text{water}}/{\left(\mu_{\text{en}}/\rho \right)}_{\text{carbon}}] \end{array}$$

(3) $$\begin{array}{l} D{\left(\text{GY}\right)}_{\text{Ppx}}=Q{\left(\text{nc}\right)}_{\text{ppx}}\times [1/\text{Sensitivity}\;\text{nC}/\text{Gy}]\times [S_{\text{ppx}}/S_{\text{ppx}}]\times \\ \;[{\left(\mu_{\text{en}}/\rho \right)}^{\text{ppx}}/{\left(\mu_{\text{en}}/\rho \right)}_{\text{carbon}}] \end{array}$$

(4) $$\begin{array}{l} D{\left(\text{GY}\right)}_{\text{Nylon}}=Q{\left(\text{nc}\right)}_{\text{nylon}}\times [1/\text{Sensitivity}\;\text{nC}/\text{Gy}]\times [S_{\text{nylon}}/S_{\text{carbon}}]\times \\ \;[{\left(\mu_{\text{en}}/\rho \right)}^{\text{nylon}}/{\left(\mu_{\text{en}}/\rho \right)}_{\text{carbon}}] \end{array}$$

**Figure 1:**
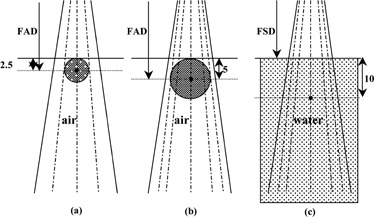
Estimation of absorbed dose in (a) nylon, (b) PMMA, and (c) water phantom.

Similar measurements were carried out with a nylon cylinder (miniphantom used for head scatter measurements, diameter 5 cm) with CC13 ion chamber and diamond detector. Absorbed dose in nylon with CC 13 was calculated by using suitable stopping power and absorption coefficients (Eq. (4)). Figure 1 shows the irradiation geometries for measurements in nylon, PMMA, and water phantoms.

Measurements were carried out with high‐energy electrons (6 Mev, 9 MeV, 12 MeV, 15 MeV, 18 MeV, and 22 MeV energies) with $15\times 15\;{\text{cm}}^{2}$ applicator using CC 13 ion chamber at depths recommended by TRS‐398 (IAEA) protocol.^(13)^ For electron beams, the value of stopping power ratios of water to graphite were taken from published tables for electron energies calculated at the reference depths ‘z’ (Ez). The effective point of measurement of the CC 13 chamber was positioned at selected depths ‘z’ which is variable, as recommended by TRS‐398 protocol. Ratio of electrons per gram of water to graphite was applied for calculations of absorbed dose for high‐energy electrons with diamond detector (Eq. (5)). All the above physical constants viz. $\left(\mu_{\text{en}}/\rho \right)$ values, stopping power ratios, electrons/gm for water, Perspex, nylon, and carbon used in Eqs. (1), (2), (3), and (4) are listed in Tables 1, 2, and 3.

(5) $$\begin{array}{l} D{\left(\text{GY}\right)}_{\text{Water}}=Q{\left(\text{nc}\right)}_{\text{water}}\times [1/\text{Sensitivity}\;\text{nC}/\text{Gy}]\times [S_{(\text{Water}.}{}_{\text{Carbon})\text{Ez}}]\times \\ \;[e^{-}{\text{density}}_{\text{water}}/e^{-}{\text{density}}_{\text{Carbon}}] \end{array}$$

**Table 3:** Electron density in media and ratios of e” /gm against carbon

$e^{-}/gm\;Water\times 10^{23}$	$e^{-}/gm\;Perspex\times 10^{23}$	$e^{-}/gm\;Nylon\times 10^{23}$	$e^{-}/gm\;Carbon\times 10^{23}$	*Ratios of Electrons/gm in Materials*		
				*water carbon*	*perspex carbon*	*nylon carbon*
3.343	3.248	3.300	3.008	1.111	1.080	1.013

### G. Calibration factors for diamond detector $N_{\text{diamond},\;\text{water}}$

**Figure 2:**
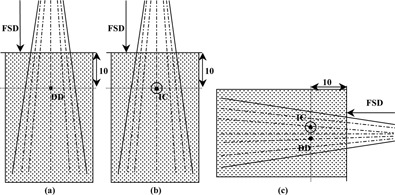
Geometry of calibration set up for diamond detector.

The methodology in the previous sections addressed the problem to derive absorbed doses from first principles. As standard cobalt‐60 beam quality is not available, we used substitution and simultaneous exposure techniques to obtain calibration factor $N_{\text{diamond},\text{water}}$ at 6 MV and 15 MV beam qualities. Blue Phantom (IBA) was used for obtaining 10 cm depth ion chamber readings and diamond detector readings and to get calibration factors by substitution. A calibration phantom of 30 × $30\times 30\;{\text{cm}}^{3}$ with a carriage carrying adopters for various chambers (IBA) was used to obtain simultaneous readings from calibrated chamber and diamond detector at both sides of central axis, and interchanging their respective positions. Ratio of readings after applying dosimetric factors was obtained for the two positions and geometric mean of the ratio provided calibration factor of the ‘diamond detector’. Geometry of calibration of diamond detector in phantom is shown in Fig. 2.

## III. RESULTS

The reproducibility in obtaining readings with diamond detector is found to be ±0.17% (1 SD) (n=11). It was observed that measurements using diamond detectors are found to be linear (see Table 4, Fig. 2). The dose rate effect observed in the microdiamond and variations in its response at change in dose rate is shown in Table 5. Δ, the sublinear dose rate parameter values calculated from obtained readings (nC) for different dose rates, are −0.00376 and −0.00310 for 6 MV and 15 MV, respectively. The response of diamond detector to scattered photon components is compared with respect to ion chamber, which is shown in Table 6. Diamond detector and ion chamber agree to have scatter factor within 1.6% and 1.1% for 6 MV and 15 MV photon beams, respectively, for a field increase of dimension 20 cm. It could be observed that the output factor of $30\times 30\;{\text{cm}}^{2}$ field size is comparatively lower in low energy linear accelerator recorded by both detectors because of the absence of additional multileaf collimator (MLC) in low‐energy linac. Comparison of diamond detector with tip of the detector facing the beam showed 2% less response compared to horizontal positioning of the detector, at desired depths.

**Table 4:** Linearity of diamond detector (60019) in Perspex phantom at 5 cm depth, 300 MU/min

	*Diamond Detector Readings (mGy)*		
∫MU	*1*	*2*	*Mean*
25	9.112	9.114	9.113
50	18.140	18.120	18.130
75	27.120	27.120	27.120
100	36.150	36.090	36.120
150	54.140	54.050	54.100
200	72.040	72.020	72.030
250	90.020	90.000	90.010
300	107.900	107.900	107.900
400	143.900	143.900	143.900

**Table 5:** Dose rate effect of microdiamond detector for linac X‐rays

	*Diamond Detector/Electrometer Readings*			*Calculated value of constant Δ*		*Constant of Proportionality*	
*Dose Rate MU/min*	*Leakage (pc)*	*6 MV nC*	*15 MV nC*	*6 MV*	*15 MV*	*6 MV*	*15 MV*
100	0.04	1.6405	1.6300				
200	0.04	1.6340	1.6250				
300	0.04	1.6310	1.6240	−0.00376	−0.003110	1.6692	1.6534
400	0.04	1.6305	1.6230				
500	0.04	1.6295	1.6215				
600	0.04	1.6295	1.6215				

**Table 6:** Field size response of diamond detector linac X‐rays

*Energy of X‐rays and Machine*	*Diamond Detector(DD)/Electrometer Readings (nC)*			*CC13/Dose 1(IC) Electrometer Readings (mGy)*			*DD/IC Scatter Factors*
	10×10	30×30	*Factor*	10×10	30×30	*Factor*	
6 MV (2300CD)	1.073	1.256	1.1705	361.3	416.2	1.1519	1.016
15 MV (2300CD)	1.226	1.358	1.1076	417.7	457.5	1.0953	1.011
6 MV (600CD)	1.047	1.196	1.1423	228.8	260.1	1.1368	1.005

### A. Absorbed dose in water for photons

Table 6 shows the comparison of diamond detector's absorbed dose estimates compared with absorbed dose estimates with Dose 1 electrometer connected to compact chamber (CC13). The absorbed dose calculated at the ‘diamond detector’ is in good agreement with measured doses in water for 6 MV and 15 MV (mean deviation −1.4% for 6 MV and −1.0% for 15 MV). The value of absorbed dose cGy/MU is without application of any other calibration factor for the diamond detector and based only on Eq. (1). Table 7 brings out the comparison of scatter factors measured by diamond detector and point ion chamber at same geometry for small fields in Perspex. It could be observed that, for fields up to 1.2਀cm×1.2 cm, the mean deviations are within 1.0%. It is noted from Table 8 that, in water at $d_{\text{max}}$ point measurements and Perspex at 5 cm depth measurements give mean deviations of about 1.5% in field scatter factors.

**Table 7:** Field size factors for mMLC (Brainlab) small fields in 6 MV X‐ray beam. (Perspex phantom, 5 cm depth, comparison of diamond detector CC 01 ion chamber (IC))

$Jaw\to Field↓MLC\;Field\;(cm^{2})$	*Detector Diam/IC*	*Jaw Opening in Clinac 600 CD (cm^2^)*							*Mean Devn. % Diam/IC*
		0.6×0.6	1.2×1.2	1.8×1.8	4.2×4.2	6.0×6.0	8.0×8.0	10.0×10.0	
0.6×0.6	Diam	0.5627	0.6725	0.6756	0.6780	0.6782	0.6791	0.6797	16.5±
	IC	0.4682	0.5782	0.5831	0.5845	0.5860	0.5870	0.5878	1.65
1.2×1.2	Diam		0.7709	0.7894	0.7976	0.7996	0.7997	0.8016	0.74±
	IC		0.7582	0.7849	0.7924	0.7961	0.7941	0.7972	0.46
1.8×1.8	Diam			0.8240	0.8473	0.8523	0.8532	0.8548	0.35±
	IC			0.8204	0.8442	0.8503	0.8506	0.8513	0.08
2.4×2.4	Diam				0.8703	0.8794	0.8833	0.8847	0.58±
	IC				0.8655	0.8749	0.8765	0.8810	0.14
3.0×3.0	Diam				0.8842	0.8967	0.9028	0.9066	0.23±
	IC				0.8815	0.8958	0.9017	0.9032	0.14
3.6×3.6	Diam				0.8968	0.9113	0.9193	0.9240	0.04±
	IC				0.8957	0.9117	0.9193	0.9233	0.07
4.2×4.2	Diam				0.9057	0.9221	0.9321	0.9377	0.16±
	IC				0.9056	0.9199	0.9316	0.9344	0.16
6.0×6.0	Diam					0.9442	0.9572	0.9660	0.09±
	IC					0.9436	0.9567	0.9646	0.06
8.0×8.0	Diam						0.9749	0.9860	0.06±
	IC						0.9733	0.9871	0.20
10×10	Diam							1.0000	**Refr**.
	IC							1.0000	**Field**

**Table 8:** Field size factors for mMLC (Brainlab) effect of phantoms. (Comparison of measured factors in water at dmax and Perspex at 5 cm depth with CC01 IC)

$Jaw\to Field↓MLC\;Field\;(cm^{2})$	*Detector Diam/IC*	*Jaw Opening in Clinac 600 CD (cm^2^)*							*Mean Devn. %*
		0.6×0.6	1.2×1.2	1.8×1.8	4.2×4.2	6.0×6.0	8.0×8.0	10.0×10.0	$W/P_{px}$
0.6×0.6	Water	0.4042	0.5963	0.6005	0.6018	0.6027	0.6208	0.6220	3.89±
	Ppx	0.4682	0.5782	0.5831	0.5845	0.5860	0.5870	0.5878	1.50
1.2×1.2	Water		0.7563	0.7946	0.8069	0.8057	0.8100	0.8110	1.27±
	Ppx		0.7582	0.7849	0.7924	0.7961	0.7941	0.7972	0.81
1.8×1.8	Water			0.8469	0.8583	0.8621	0.8639	0.8654	1.88±
	Ppx			0.8204	0.8442	0.8503	0.8506	0.8513	0.74
2.4×2.4	Water				0.8784	0.8862	0.8902	0.8936	1.44±
	Ppx				0.8655	0.8749	0.8765	0.8810	0.11
3.0×3.0	Water				0.8944	0.9059	0.9128	0.9164	1.32±
	Ppx				0.8815	0.8958	0.9017	0.9032	0.17
3.6×3.6	Water				0.9039	0.9192	0.9274	0.9332	0.95±
	Ppx				0.8957	0.9117	0.9193	0.9233	0.16
4.2×4.2	Water				0.9117	0.9286	0.9386	0.9453	0.87±
	Ppx				0.9056	0.9199	0.9316	0.9344	0.24
6.0×6.0	Water					0.9472	0.9623	0.9723	0.59±
	Ppx					0.9436	0.9567	0.9646	0.21
8.0×8.0	Water						0.9760	0.9905	0.10±
	Ppx						0.9733	0.9871	0.34
10×10	Water							1.0000	**Refr**.
	Ppx							1.0000	**Field**

### B. Calibration factors N_diamond,water_

Using nominal sensitivity of 1 nC/Gy, the absorbed dose estimates for 6 and 15 MV photons with diamond detector compared with calibrated ion chamber is shown in Table 9. The calibration factors obtained in water to derive absorbed doses using diamond detector have magnitude of deviations matching with water measurements, as shown in Table 10.

**Table 9:** Measurements in water phantom with photons

		*Absorbed Dose (cGy/MU)*		
*No*.	*MV X‐ray Machine*	*IC CC13/Dose1* d=10 cm	*Diamond/Tandem* d=10 cm	*% SD*
1	6 MV	0.6932	0.6823	−1.6%
	15 MV	0.7872	0.7833	−0.5%
	2300 CD			
2	6 MV	0.6932	0.6722	−3.2%
	15 MV	0.7856	0.7737	−1.5%
	2300 CD			
3	6 MV	0.6694	0.6558	−2.0%
	600 CD			

**Table 10:** Calibration factors for ‘diamond detector’ against ion chamber N_D,water_

*Photon Energy MV*	*Substitution Method*			*Simultaneous Exposure Method*		
	*Dose 1*	*Diamond*		*Factor*	*Factor*	*Factor*
	*cGy/MU*	*cGy/MU*	*Factor*	*Posn. 1,2*	*Posn. 2,1*	*Geom.Mean*
6 MV Clinac 2300	1)0.6932	1)0.6823	1.0160	$\frac{0.6779}{0.6778}$	$\frac{0.6845}{0.6656}$	1.0142
	2)0.6932	2)0.6722	1.0312	=1.000	=1.0284	
15 MV Clinac 2300	1)0.7872	2)0.7833	1.0050	$\frac{0.7793}{0.8081}$	$\frac{0.8014}{0.7780}$	0.9963
	2)0.7873	2)0.7737	1.0175	=0.9644	=1.0301	
6 MV	1)0.6694	1)0.6558	1.0207	1)$\frac{0.6639}{0.6501}$	$\frac{0.6618}{0.6514}$	1.0186
				=1.0212	=1.0160	
Clinac 600				2)$\frac{0.6272}{0.6120}$	$\frac{0.6243}{0.6140}$	1.0191
				=1.0248	=1.0135	

### C. Absorbed doses in Perspex (PMMA) phantom for photons

The measured absorbed doses in Perspex by FC 65 and CC 13 ion chambers and diamond detector are shown in Table 11. The measured absorbed doses are in agreement with the calibrated ion chambers within ±2.0%.

**Table 11:** Measurements in Perspex tool in linac X‐rays

			Diamond Detector Dose	
*No*	*Machine/MV X‐ray*	*lon Chamber Dose cGy/MU*	*cGy/MU*	*Deviation*
1	600 CD	0.8704	0.8557	−1.7%
	6 MV	FC 65)		
2	2300 CD			
	6 MV	0.8948	0.8816	−1.5%
	15 MV	0.9632	0.9835	+2.1%
		(FC 65)		
3	2300 CD			
	6 MV	0.8946	0.8806	−1.5%
	15 MV	0.9624	0.9639	+0.2%
4	2300 CD	(CC13)		
	6 MV	0.8956	0.8757	−2.2%
	15 MV	0.9623	0.9597	−0.3%
		(CC13)		
5	600 CD			
	6 MV	0.8683	0.8510	−2.0%
		(CC13)		

### D. Absorbed doses in nylon phantom for photons

The comparison of measured absorbed doses of diamond detector with Dose 1 ion chamber measurements in nylon miniphantom is shown in Table 12. The cGy/MU dose estimate deviation in diamond detector was within 3.5% for 6 MV beam and within 2.0% for 15 MV beam.

**Table 12:** Measurements in nylon miniphantom in linac X‐rays

			*Diamond Detector Dose*	
*No*	*Machine/MV X‐ray*	*IC Dose cGy/MU*	*cGy/MU*	*Deviation*
1	6 MV	1.0003	0.9836	−1.7%
	15 MV 2300 CD	1.0482	1.0460	−0.2%
2	6 MV	1.0210	0.9870	−3.4%
	15 MV 2300 CD	1.0625	1.0413	−2.0%
3	6 MV 600 CD	0.9675	0.9493	−1.9%

### E. Absorbed dose in water for high‐energy electron beams

Table 13 shows the absorbed dose estimates in high‐energy electrons with water phantom. From 12 MeV to 22 MeV electrons, diamond detector measured absorbed doses within 3% agreement. For a couple of electron beams, the measured absolute doses are more than 3% different from ionization chamber measurements. Ion chamber was placed with its center at the effective point of measurement giving an offset in positioning. Comparison of diamond detector response at its physical center, as well as by shifting centre to effective point of measurement, did not show significant changes in readings.

**Table 13:** Measurements in water phantom with high‐energy electrons

	*Electron Energy MeV/Depth mm*	*IC Dose cGy/MU (CC13)*		*Diamond Detector Measured Dos*			
				*cGy/MU*	*cGy/MU*	*Deviation in Dose*	
*No*.		*1*	*2*	*Not eff.pt*.	*At eff. pt*	*Not eff.pt*.	*At eff. pt*
1	6 MeV/ 14.0	1.0814	1.0875	1.0056	0.9799	−7.0%	−8.9%
2	9 MeV/ 21.2	1.0290	1.0343	0.9909	0.9656	−6.7%	−6.7%
3	12 eV/29.4	1.0438	1.0485	1.0155	0.9990	−2.7%	−4.8%
4	15 eV/37.3	1.1279	1.1351	1.0968	1.0955	−2.8%	−3.5%
5	18 eV/45.0	1.0701	1.0777	1.0498	1.0502	−1.9%	−2.6%
6	22 eV/52.2	0.9992	1.0128	0.9896	0.9965	−1.0%	−1.6%

## IV. DISCUSSION & CONCLUSION

The present work illustrates our implementation of microdiamond detector (TM 60019) for absorbed dose measurements in the department. This detector is the first of its design, fabricated from M/s PTW, Germany. This has bias voltage kept at V=0 and, therefore, there is no influence of applied voltage between colleting electrodes, therefore the signal representing the total charge released by photoconduction. If we assume that the released charge (nC) represent the total amount of ionization in ‘diamond’ (which is an allotropic form of carbon), then from first principles, we would be able to realize absorbed dose by substituting relevant radiation physics quantities.

Our results of absorbed dose measured in 6 MV and 15 MV photons with diamond detector, in water, nylon and Perspex, and comparison with calibrated thimble chamber measured doses, have shown that this diamond detector (TM 60019) can measure absorbed dose in a medium, as an absolute dosimeter. In nylon and Perspex miniphantoms, the deviations are slightly more compared to water phantom measurements. The explanation is that the diamond detector's tip does not exactly fit the position of CC 13 chamber because CC 13 has rounded tip, whereas diamond detector has flat edge.

It is felt that, if the calibration factor obtained for 1.02 for 6 MV and 1.006 for 15 MV by our measurements (Table 7) are applied to the absorbed dose in water by ‘diamond’, there will be better agreement with ion chamber values. While discussing the effect of field size on lateral electronic equilibrium in small fields, an energy spectrum correction factor of 1.016 for 6 MV and 1.012 for 10 MV was proposed for diamond detector by Sauer and Wilbert.^(14)^ In another report, small field output factors up to a field size of $0.8\times 0.8\;{\text{cm}}^{2}$ were acquired using PTW 60003 diamond detector in Elekta linear accelerator.^(15)^ Therefore, it becomes clear that there is a definite role of diamond detectors in quantifying absorbed doses in small fields.

In electron beams, the observed deviations with respect to CC 13 chamber were more, especially in energies up to 12 MeV. This may be due to the positioning inaccuracy of diamond detector (which has effective size of radius 1.1 mm, compared to compact chamber dimension of 0.13 cc, with external diameter of 6.8 mm. Microdiamond detector (type 60019) used in this study has a sensitive volume 0.004 mm^3^ and thickness 1 μm, compared to earlier version of 60003.^(5)^ In model 60003, of a natural diamond crystal type, the sensitive area is 6.8 mm^2^ and thickness is 0.25 mm, giving a sensitive volume of 1.7 mm^3^. This is an earlier version to microdiamond type, which is operated at +100 volts, and has higher nC/Gy. Characterization of CVD diamond crystals to configure radiation dose patterns had been reported in many earlier works. They had thickness varying from 100 to 500 μm, sensitivities ranging from 3 to 178 nC/ Gy, and variable operating voltage bias. These reports did not mention the use of ‘diamonds’ for measuring absolute dose. Our present work therefore assumes importance.

In confirmation of dose in segmental fields of IMRT, Barnett et al.^(5)^ brought up an important point for consideration. They reported that, because diamond detector is of negligible dimensions compared to ion chambers and solid‐state detectors, sometimes more deviations could be observed with diamond detectors in high‐dose gradient regions. At improved detector position, agreement between measured doses with PinPoint chamber and the EPID within one standard error and well within 1.5% was reported. Their argument clearly reinforced the accuracy of diamond detector in measuring absorbed dose in a tissue‐equivalent medium. An earlier report from a radiation laboratory^(16)^ has brought out the efficacy of diamond detectors, with following characteristics: signal stability within 0.5% achieved by pre‐irradiation about 2 Gy radiation dose; long‐term stability in response to cobalt‐60 beam quality, within 0.4% over a period of 15 months; linearity index equal to unity within 0.2% and dark current correction within 0.2%. The detector response is independent of field size from $10\times 10\;{\text{cm}}^{2}$ fields down to $2\times 2\;{\text{cm}}^{2}$. $D_{w}$ values measured with diamond detectors for field sizes as small as $1\times 1\;{\text{cm}}^{2}$ agreed with alanine dosimeter within 1% of dose value. TM 60019 has a 0.004 mm^3^ volume, but a 4 mm^2^ active area since it is only 1 micrometer thick. PTW's TM 31016 PinPoint chamber has a volume of 0.016 mm^3^ but a 6 mm^2^ active area. When the measurements are carried out with diamond detector with its axis perpendicular to the beam, the 1 μm dimension only becomes relevant to the radiation measurement. When these two detectors are compared in profile scans, very little difference can be detected in a small field's penumbra. This idea is important when comparing diodes and diamond detectors since their volumes might be comparable but the diode will have a much smaller cross‐sectional area. With all above characteristics, Pimpinella et al ^(16)^ opined that diamond dosimeters qualify as transfer standards. In the above background, our present work supports the application of microdiamond detector for pretreatment quality assurance of treatment plans in special relevance to small field radiotherapy.

## COPYRIGHT

This work is licensed under a Creative Commons Attribution 4.0 International License.

## Supporting information

Supplementary Material Files
