# Supplementary material for: Diamond detector in absorbed dose measurements in high‐energy linear accelerator photon and electron beams
Source: J Appl Clin Med Phys. 2016 Mar 8;17(2):291–303. doi: 10.1120/jacmp.v17i2.5690 (PMC5875569; doi:10.1120/jacmp.v17i2.5690)
Supplement: Supplementary file 1 — Supplementary Material Files [file ACM2-17-291-s001.doc]

**Diamond detector in absorbed dose measurements in high energy linear accelerator photon and electron beams**

Ravichandran .R,

Binukumar .J.P,

Iqbal Al Amri,

Davis.C.A

Medical Physics Unit, Department of Radiation Oncology,

National Oncology Center, Royal Hospital, Muscat, Oman

Address for correspondence:

**Prof.Dr.Ramamoorthy Ravichandran,**

**Medical Physics Unit,**

**Department of Radiation Oncology,**

**National Oncology Centre,**

**Royal Hospital,**

**PBox 1331, PC 111,**

**Muscat, Sultanate of Oman.**

**Ph: 00968-2462 7003**

**Fax: 00968-2462 7004**

**Email: ravichandranrama@rediffmail.com**

**Acknowledgements:** Authors thank Director General, Royal Hospital for kind permission obtained for the study.

**Conflicts of Interests:** There is no conflicts of interests expressed by the authors or any other agency.

**Diamond detector in absorbed dose measurements in high energy linear accelerator photon and electron beams**

Abstract

Diamond detectors (DD) are preferred in small field dosimetry of radiation beams, because of small dose profile penumbras, better spatial resolution, and tissue equivalent properties. We investigated a commercially available ‘micro diamond’ detector in realizing absorbed dose from first principles.

A microdiamond detector, type TM 60019 (PTW, Freiburg) with tandem electrometer is used to measure absorbed doses in water, nylon and PMMA phantoms. With sensitive volume 0.004mm^3^, radius 1.1mm, thickness 1μm, the nominal response is 1nC/Gy. It is assumed that the diamond detector could collect total electric charge (nC) developed during irradiation at 0 V bias. We found that dose rate effect is less than 0.7% for changing dose rate by 500 MU/min.

The measured absorbed doses for 6 MV and 15 MV photons arrived at using mass energy absorption coefficients and stopping power ratios compared well with Nd, water calibrated ion chamber measured absorbed doses within 3% in water, PMMA and nylon media. The calibration factor obtained for diamond detector, confirmed response variation is due to sensitivity due to difference in manufacturing process. For electron beams, we had to apply ratio of electron densities of water to carbon.

Our results qualify diamond dosimeter as a transfer standard, based on long term stability, reproducibility. Based on micro-dimensions we recommend these detectors, for pre-treatment dose verifications in small field irradiations like stereotactic treatments with image guidance.

**Key words: diamond detector, small field RT, absorbed doses, stereotactic radiotherapy**
